# Accessing the SEED Genome Databases via Web Services API: Tools for Programmers

**DOI:** 10.1186/1471-2105-11-319

**Published:** 2010-06-14

**Authors:** Terry Disz, Sajia Akhter, Daniel Cuevas, Robert Olson, Ross Overbeek, Veronika Vonstein, Rick Stevens, Robert A Edwards

**Affiliations:** 1 Mathematics and Computer Science Division, Argonne National Laboratory, Argonne, IL 60439, USA; 2 Computation Institute, University of Chicago, Chicago, IL 60637, USA; 3 Computational Sciences Research Center, San Diego State University, San Diego, CA 92182, USA; 4 Fellowship for the Interpretation of Genomes, Burr Ridge, IL, 60527, USA; 5 Department of Computer Science, San Diego State University, San Diego, CA 92182, USA

## Abstract

**Background:**

The SEED integrates many publicly available genome sequences into a single resource. The database contains accurate and up-to-date annotations based on the subsystems concept that leverages clustering between genomes and other clues to accurately and efficiently annotate microbial genomes. The backend is used as the foundation for many genome annotation tools, such as the Rapid Annotation using Subsystems Technology (RAST) server for whole genome annotation, the metagenomics RAST server for random community genome annotations, and the annotation clearinghouse for exchanging annotations from different resources. In addition to a web user interface, the SEED also provides Web services based API for programmatic access to the data in the SEED, allowing the development of third-party tools and mash-ups.

**Results:**

The currently exposed Web services encompass over forty different methods for accessing data related to microbial genome annotations. The Web services provide comprehensive access to the database back end, allowing any programmer access to the most consistent and accurate genome annotations available. The Web services are deployed using a platform independent service-oriented approach that allows the user to choose the most suitable programming platform for their application. Example code demonstrate that Web services can be used to access the SEED using common bioinformatics programming languages such as Perl, Python, and Java.

**Conclusions:**

We present a novel approach to access the SEED database. Using Web services, a robust API for access to genomics data is provided, without requiring large volume downloads all at once. The API ensures timely access to the most current datasets available, including the new genomes as soon as they come online.

## Background

At least 1,000 genomes have now been sequenced and released to the public, the vast majority of which are microbial genomes. For example, the SEED currently contains over 850 Bacterial genomes that have been completely sequenced (Table 1; The SEED also contains many hundreds of draft genomes (those that are in many contigs and whose sequencing status is in flux).) For several years now it has been realized that the most efficient and accurate way of annotating these genomes is not by considering each in isolation, but by comparing them all together in unified integration platforms [1]. The SEED http://www.theseed.org/ contains all publicly available genome sequences. The underlying set of databases includes functional annotations, subsystems [2], and EC, reaction [3], and GO terms [4] for proteins in all microbial genomes. The database also houses precomputed "all-versus-all" BLAST comparison of a non-redundant database (all non-redundant proteins from all of the genomes were compared to each other using BLAST), functional coupling data that describes genes that are linked together based on homologs in other genomes, links to other data resources, and so on [5].

**Table 1:** Genomes in the SEED database as of November 26^th^, 2009.

*Domain*	*Complete*	***Draft***^*1*^
Bacteria	872	82

Archaea	52	7

Eukaryota	29	32

^1^Draft genomes are incompletely assembled and contain more than 100,000 bp of sequence.

**Figure 1:**
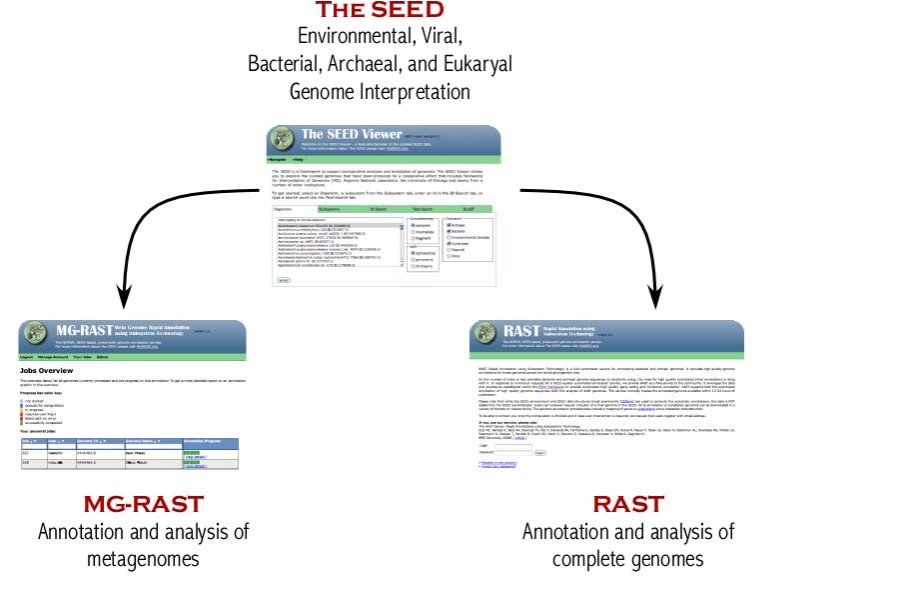
**Overview of the SEED family of services**. Each member of the family contributes a unique service to microbial genome analysis. The underlying platform, the SEED, integrates complete microbial genomes and data associated with them. The RAST server provides automatic high-quality annotation of complete genomes, while the mg-RAST server provides automatic high-quality annotation of metagenomes.

The SEED platform provides the underpinnings to several common microbial genome annotation services (Fig.1). The Rapid Annotation using Subsystem Technology (RAST server) provides high throughput accurate annotations for complete microbial genomes [6,7]. The development of the RAST server for complete microbial genome annotation provides consistent and accurate annotations, automatic connections to metabolic reconstructions, and detailed comparative genomics tools previously only available in limited environments. The metagenomics-RAST produces high throughput annotations of random community genomes [8]. The development of an annotation pipeline for random community genomics (metagenomics) has opened the field to researchers, providing high-performance bioinformatics analysis previously only available to researchers with dedicated compute power. Together the SEED family are more than just databases, as they include all the data, the access methods, and the encodings. They are open source software, freely available to all researchers, and there are no restrictions on their use. They are frequently updated as new microbial genomes are released to the public, and annotated via the RAST system [2,5,7].

Since their inception by the Fellowship for Interpretation of Genomes (FIG), these tools were built around an open-source framework that encourages development of new tools and ideas. Although the primary servers are maintained at Argonne National Laboratory and the University of Chicago, several remote SEED installations have been provided for groups requiring programmatic access to the SEED data. However, the main difficulty with remote installations is the maintenance and constant updates that are required, often beyond the capability of the average bioinformatics group. A series of Web services has therefore been developed to provide an API to the annotations of microbial genomes without requiring any downloads or installation.

SOAP services are available from EBI [9,10], KEGG [11,12], and NCBI [13,14]. The existing methods were taken into consideration when developing the SEED Web services interface and our aim is to provide compatible services. However, as further web service APIs are developed, a common set of methods, or a thesaurus to compare methods, should be defined to ensure maximum compatibility and computability between services.

To aid programmatic access to the SEED family of services, an application programming interface (API) was developed based on the Simple Object Access Protocol (SOAP) standard. Here, we describe the basic implementation of the API, and provide example code to query the databases.

### Implementation

The Web services are implemented as a Perl abstraction to the SEED database on the remote server, however the distal implementation does not limit the user's choice in language or implementation methods. The examples shown here include Perl, Python, and Java, and many other programming languages support SOAP allowing the user to choose their favourite language for their implementation.

## Results

Before the services are described, a couple of formalities about the underlying SEED database are introduced. These are provided to orient new users of the database.

### Internal Identifiers

The SEED family of databases and services has their own internal identifiers, called FIG identifiers (FIDs), in the format fig|xxxxx.i.type.yyyy. In this representation, the fig| denotes that it is a FIG internal identifier, the xxxxx is usually the NCBI taxon ID of the genome, the .i is the increment of the genome (advanced when major changes are performed), the type is the feature type, and the yyyy is the number of the feature on the genome. Feature types are typically peg (*p*rotein *e*ncoding *g*ene), rna, pp (prophage), pi (pathogenicity island), and so on. The feature type is lower case, and the number is usually incremented along the chromosome. However, features that are inserted will get the next available, unused, feature number, and the numbers from deleted features are not recycled. Therefore although features with adjacent numbers are usually adjacent to each other on the chromosome, that is not guaranteed.

Thus, "fig|243277.1.peg.4400" refers to the 4400^th ^protein encoding gene in the 1^st ^increment of the genome with taxonomy ID 243277 (*Vibrio cholerae *O1 biovar eltor str. N16961). The functional annotation of this protein is "β-subunit of the DNA-directed RNA polymerase (EC 2.7.7.6)". The identifier "fig|243277.1.rna.23" refers to the 23^rd ^RNA feature of the same genome. For simplicity, these two examples are used throughout this discussion. The genome identifier in this case is 243277.1 (note that we include the increment number with the taxonomy identifier). For access to web pages and user controlled material, the link-in URLs based on http://www.theseed.org/linkin.cgi provide access to pages related to the genome, proteins, subsystems and associated data. For example, http://www.theseed.org/linkin.cgi?genome=243277.1 links to the organism overview for *Vibrio cholerae *O1 biovar eltor str. N16961, and http://www.theseed.org/linkin.cgi?id=fig|243277.1.peg.4400 links to the page related to the protein sequence.

### External Identifiers

In addition to these internal identifiers, the SEED database maintains mapping to other commonly used identifiers wherever possible. For example, the peg shown above also has the following aliases: GeneID:2615094 NP_229982.1 VC0328 gi|15640355 gi|41019520 kegg|vch:VC0328 sp|Q9KV30 uni|Q9KV30. Typically, the source database is abbreviated, precedes the identifier, and is separated from the identifier with a vertical bar (e.g. sp is SwissProt and uni is UniProt).

### Accessing the SEED via Web services

The Web services API provides ready access to commonly used methods to retrieve sequence and related data from the underlying database. An appropriate Web services description language XML (WSDL) file must be retrieved to discover which services are available. This file contains required information about each call, and informs the SOAP client of URLs and namespaces for the servers, procedures that are available, and parameters required for those calls. WSDL files are often generated statically, and have to be updated to reflect changes to the API. In contrast, the SEED WSDL files are dynamically generated from the publicly exposed methods at the time of calling, and thus the files are constantly current and updated as new methods are exposed. The currently available list of methods that can be used for Web service calls, their input parameters, and their output strings are shown in Table 2.

**Table 2:** Methods, input and output parameters in the SEED Web services API

*Method Name*	*Parameters & Order*	*Description*
abstract_coupled_to	peg	Get the pegs that may be coupled to this peg through abstract coupling. Input is a peg, output is list of [protein, score] for things that are coupled to this peg

Adjacent	pegs	Retrieve the set of pegs in order along the chromosome. Input is a comma separated list of pegs, and output is the pegs in order along the genome.

alias2fig	alias	Get the FIG ID(s) (peg) for a given external identifier. Input is an identifier used by another database, output is a list of our identifiers. Note that an alias can refer to more than one protein since the mapping is done via protein sequence.

aliases_of	peg	Get the aliases of a peg. These are the identifiers that other databases use. Input is a peg, output is an array of aliases

ali_to_seq	alias	Retrieve the protein sequence for a given identifier. Input is an alias, output is a sequence

all_families		Get all the FIG protein families (FIGfams). No input needed, it just returns a list of all families

all_families_ with_funcs		Get all the FIG protein families (FIGfams) with their assigned functions. No input needed, it just returns a list of all the families and their functions.

all_genomes	complete, restrictions, domain	Get a set of genomes. The inputs are a series of constraints - whether the sequence is complete, other restrictions, and a domain of life (Bacteria, Archaea, Eukarya, Viral, Environmental Genome). Output is a list of genome ids. An example use is with the parameters ("complete", undef, "Bacteria") that will return all complete bacterial genomes.

all_subsystem_ classifications		Get a list of all the subsystems and their classifications. No input needed, it just returns a list of all the subsystems and their classifications

boundaries_of	locations	Get the boundaries of a feature location. A feature can have multiple locations on a contig (e.g. split locations, introns, etc). This just returns an array of [contig, beginning, end]. You can pass it the output from feature_location directly

CDS_data	families	Get all the pegs in some FIGfams, their functions, and aliases. Input is a tab-separated list of pegs, returns a 3-column comma separated table [peg, Function, Aliases]

CDS_sequences	families	Get the protein sequences for a list of proteins. Input is a tab-separated list of peg, returns a 2-column comma separated table of [peg, sequence]

cluster_by_bbhs	peg	Get the clusters for a peg by bidirectional best hits. Input is a peg, output is two column table of [peg, cluster]

cluster_by_sim	peg	Get the clusters for a peg by similarity. Input is a peg, output is two column table of [peg, cluster]

contigs_of	genomeid	Get a comma-separated list of all the contigs in a genome

contig_ln	genomeid, contig	Get the length of the DNA sequence in a contig in a genome. Input is a genome id and a contig name, return is the length of the contig

coupled_to	peg	Get the pegs that are coupled to any given peg. Input is a peg, output is list of [protein, score] for things that are coupled to this peg

dna_seq	genomeid, location1	Get the DNA sequence for a region in a genome. Input is a genome ID and a location in the form contig_start_stop, output is the DNA sequence in fasta format.

ec_name	EC_number	Get the name for a given E.C. number. Input is an EC number, output is the name

external_calls	peg	Get the annotations for a peg from all other known sources. Input is a peg, output is two column table of [peg, other function]

feature_location	peg	Get the location of a peg on its contig. Input is a peg, output is list of locations on contigs. Usually this will be a single location, but sometimes it can either be more than one region on a contig, or even on multiple contigs. For convenience it is a comma joined list, often you will want to pass that to boundaries_of

fid2dna	peg	Get the DNA sequence for a given protein identifier. Input is a peg, output is the DNA sequence in fasta format.

fids2dna	peg	Get the DNA sequence for a set of protein identifiers. Input is a comma-joined list of pegs, output is the DNA sequence in fasta format.

function_of	peg	Get the functional annotation of a given protein identifier. Input is a peg, output is a function

Genomes	complete, restrictions, domain	Get a set of genomes. The inputs are a series of constraints - whether the sequence is complete, other restrictions, and a domain of life (Bacteria, Archaea, Eukarya, Viral, Environmental Genome). Output is a list of genome ids with the genus species appended. An example use is with the parameters ("complete", undef, "Bacteria") that will return all complete bacterial genomes.

genomes_of	peg	Get the genome(s) that a given protein identifier refers to. Input is a peg, output is a single column table of genomes

genus_species	genomeid	Get the genus and species of a genome identifier. Input is a genome ID, output is the genus and species of the genome

get_ corresponding_ ids	peg	Get the corresponding ids of a peg. These are the identifiers that other databases use. Input is a peg, output is an array of aliases

get_dna_seq	featureid	Retrieve the DNA sequence for a particular feature. Note that this will take a feature id (peg, rna, etc), and return the DNA sequence for that id. There is also a separate method to get the DNA sequence for an arbitrary location on a genome

get_translation	peg	Get the translation (protein sequence) of a peg. Input is a peg, output is translation. (Note that this is a synonym of translation_of);

is_archaeal	genomeid	Test whether an organism is Archaeal. Input is a genome identifier, and output is true or false (or 1 or 0)

is_bacterial	genomeid	Test whether an organism is Bacterial. Input is a genome identifier, and output is true or false (or 1 or 0)

is_eukaryotic	genomeid	Test whether an organism is Eukaryotic. Input is a genome identifier, and output is true or false (or 1 or 0)

is_member_of	sequences	Tries to put a protein sequence in a family. Input is a tab-separated id and sequence, delimited by new lines. The output is a comma-separated 2-column table [your sequence id, FamilyID] if the sequence is placed in a family.

is_prokaryotic	genomeid	Test whether an organism is a Prokaryote. Input is a genome identifier, and output is true or false (or 1 or 0)

list_members	families	Get all the pegs in some FIGfams. The input is a tab-separated list of family IDs, and the output is a two column table of [family id, peg]

pegs_of	genomeid	Get all the protein identifiers associated with a genome. Input is a genome id, output is a list of pegs in that genome

pegs_with_md5	md5	Get the FIG IDs associated with the MD5 sum of a protein sequence. Input is the md5 checksum, output is an array of strings of FIG ids. This should be faster, and more complete, than using aliases or other ways to match protein sequences.

pegs_with_md5_string	md5	Get the FIG IDs associated with the MD5 sum of a protein sequence. Input is the md5 checksum, output is a comma separated list of FIG ids as a single string. This should be faster, and more complete, than using aliases or other ways to match protein sequences.

pinned_region_ data	peg_id, n_pch_pins, n_sims, sim_cutoff, color_sim_ cutoff, sort_by	Input is a FIG (peg) ID and ..., output is the pinned regions data

reaction_to_role	Reaction_number, genomeid	Get a tab-separated list of [subsystem name, functional role, peg, subsystem variant code for that genome] for any given reaction id and genome id. Maps the reaction id to peg, peg to genome, and genome to variant code

replaces	genomeid	If this genome replaces another one (it is a more upto date version), what is the ID of the older genome?

Rnas_of	genomeid	Get all the RNA identifiers associated with a genome. Input is a genome ID, and output is a list (an array) of the RNAs in that genome

search_and_grep	pattern1, pattern2	Search and grep through the database. Input is two patterns, first one is used in search_index, second used to grep the results to restrict to a smaller set. Output is an array of hashes with keys id, organism, otherIds, functionalAssignment, and annotator.

Simple_search	pattern	Search the database. Input is a pattern to search for, output is list of pegs and roles

Sims	peg, maxN, maxP	Retrieve the sims (precomputed BLAST hits) for a given protein sequence. Input is a peg, an optional maximum number of hits (default = 50), and an optional maximum E value (default = 1e-5). The output is a list of sims in modified tab separated (-m 8) format. Additional columns include length of query and database sequences, and method used.

taxonomy_of	genomeid	Returns the taxonomy of a given genomeid

translation_of	peg	Get the translation (protein sequence) of a peg. Input is a peg, output is the protein sequence. (Note that this is a synonym of get_translation).

The examples discussed below all use Perl http://www.perl.org/ and the SOAP::Lite Perl module available from http://search.cpan.org/. In the examples below we use the simple SOAP::Lite interface making HTTP calls via port 80. This will be sufficient for most API calls, and more details about the SOAP::Lite interface can be found online or in the O'Reilly *Programming Web Services With Perl *[15]. Python and Java code that works with the Web services interface is included in the online examples.

To initiate a connection using Perl and SOAP::Lite, the constructor SOAP::Lite->service is provided the URL for the publicly available WSDL file. The dedicated Web services server machine at http://ws.theseed.org/ is optimized for handling Web services calls rather than user-initiated calls (Code 1 in the Additional File 1). The constructor generates method stubs that can then be called as methods of the service. Most commonly used methods are described below.

### Searching the SEED

To search the SEED, two different access methods are provided. The *simple_search *accepts a query string, and returns all data that matches the string. For example, searching for "VC0328" returns the text separated by tabs as shown in Code 2 in the Additional File 1.

The first item is the internal identifier, and the second the genome from which it came. The third item is a list of all other aliases for this peg. The alias list is constructed based on sequence identity [5]. Fourth is the functional annotation of the protein, and the last item is the person that made the annotation - in this case a master (or trusted) annotator made the annotation.

The second method provided for searching the SEED is via *search_and_grep*. This method takes two arguments, the first is what to search for, and the second is a regular expression that should be found within the search string. This provides a server-side mechanism for reducing the output of the search. For example, a *simple_search *for *dnaA *returns 2,774 items, but a *search_and_grep *for "dnaA" and "Vibrio" reduces the list to 56 items (the grep is always case sensitive).

### Working with genomes

To retrieve all the genomes in the current instantiation of the SEED database, a call to *genomes *is made. This method takes three optional constraints, the first is a boolean, if true only "complete" genomes will be returned, if false all genomes will be returned. The second, is a set of restrictions that can be applied on a genome-by-genome basis, and the third option is a domain to return (Bacteria, Archaea, Eukarya, or Environmental Sample). The block of code shown in Code 3 in the Additional File 1 returns all complete Bacterial genomes in the SEED. The same code is shown in Java, Python, and Perl to demonstrate the portability of the Web services approach.

The returned data is an array of tuples of [genome ID, genome name] separated by a tab. Additionally, the genome name can be retrieved using the *genus_species *call, that accepts a genome ID as its sole argument.

For any genome ID, every protein encoding gene (peg) of the genome can be retrieved by using the call *pegs_of*. This simply returns a list of FIDs in each genome that can be parsed using the methods described below. As noted above, the pegs are typically in numerical order along the chromosome but that is not guaranteed as pegs may be added to fill in missing genes. The method *adjacent *takes a list of pegs and sorts them in order along the chromosome. Thus, the combined call shown in Code 4 in the Additional File 1 will return a list of ordered pegs. Of course, as shown below, the location of each peg can be retrieved and sorted locally by the user, if desired.

### Working with genes and proteins

Many methods are available to retrieve the data underlying the SEED, and most work at the level of the protein. As noted above, both internal and external identifiers are maintained, but typically API requests are made with internal identifiers (FIDs), as shown here. Simple functional calls include the ability to retrieve the location of a FID on a contig, the DNA or protein sequence, the annotation, as shown in Table 2.

For example the block of code shown as Code 5 in the Additional File 1 will retrieve the location of the sequence (contig, start position and stop position), and the protein sequence of the peg from *Vibrio cholerae. *The protein sequence is in fasta format, suitable for feeding into other bioinformatics applications. Similarly, the *fid2dna *method returns the DNA sequence in fasta format.

An underlying resource in the SEED database is the precomputed coupling of proteins along and between genomes [1]. Coupling is an evidence-based metric of the co-occurrence of any pair of proteins in unrelated genomes, and infers that proteins are involved in the same cellular process. Coupling evidence is one of the pieces of information SEED annotators use to infer function. Two methods are currently provided to return coupling data. First, *coupled_to *takes a given peg and returns a list of pegs that it is coupled to, along with a normalized score for that coupling [2]. The score is the number of genomes in which similar coupling is retained in nearby pegs.

The second method, *abstract_coupled_to *is related to the coupling. Coupling requires that two genes co-occur near each other on two genomes, however we realized that sometimes genes co-occur but are not next to each other. Abstract coupling does not require that two genes be adjacent in a genome if there is evidence from other genomes that suggests that the genes are adjacent. This "abstract evidence" can be used to assert related functions. As shown in Table 3, the direct coupling shows genes that are related to fig|243277.1.peg.4400, both in terms of location and function. The abstract coupling identifies the same related genes, but also identifies near neighbors that are implicated by distant genomes, but are not neighbors in *V. cholerae *N16961.

**Table 3:** Pegs that are coupled to fig|243277.1.peg.4400 either directly through close association, or in an abstract manner

*Peg*	*Coupled Score*	*Abstract Coupling Score*	*Function*
fig|243277.1.peg.316	6	0.38	Translation elongation factor Tu

fig|243277.1.peg.318	39	0.65	Transcription antitermination protein NusG

fig|243277.1.peg.319	24	0.61	LSU ribosomal protein L11p (L12e)

fig|243277.1.peg.320	36	0.64	LSU ribosomal protein L1p (L10Ae)

fig|243277.1.peg.321	11	0.58	LSU ribosomal protein L10p (P0)

fig|243277.1.peg.322	25	0.67	LSU ribosomal protein L7/L12 (L23e)

fig|243277.1.peg.324	34	0.64	DNA-directed RNA polymerase beta' subunit

fig|243277.1.peg.354	-^1^	0.42	SSU ribosomal protein S12p (S23e)

fig|243277.1.peg.355	-	0.5	SSU ribosomal protein S7p (S5e)

fig|243277.1.peg.4033	-	0.35	Preprotein translocase subunit SecE

fig|243277.1.peg.356	-	0.27	Translation elongation factor G

^1^These proteins are not coupled directly.

The SEED contains precomputed similarities for all proteins compared to all other proteins in the database. This is maintained essentially as the tabular output from NCBI BLASTALL [16] appended with the length of the query protein and the length of the database protein and the method used to identify the similarity. The *sims *method takes a peg and returns everything that is similar to it, within the optional limits provided by the user. Two limits are supported, the maximum number of similarities returned and the maximum expect (E) value for the sims. Thus, when executed the code shown in Code 6 in the Additional File 1 returns the output shown in Table 4.

**Table 4:** Similarities returned for fig|243277.1.peg.4400

Query	Database	P	L	G	M	QS	QE	DS	DE	E	BS	QL	DL	Me
fig|243277.1.peg.4400	fig|345072.3.peg.508	100.00	1341	0	0	1	1341	1	1341	0	2682	1341	1375	blastp
fig|243277.1.peg.4400	fig|345075.3.peg.501	100.00	1341	0	0	1	1341	1	1341	0	2682	1341	1375	blastp
fig|243277.1.peg.4400	fig|404974.3.peg.2938	100.00	1341	0	0	1	1341	1	1341	0	2682	1341	1375	blastp
fig|243277.1.peg.4400	fig|412614.3.peg.2528	100.00	1341	0	0	1	1341	1	1341	0	2682	1341	1375	blastp
fig|243277.1.peg.4400	fig|412966.3.peg.2460	100.00	1341	0	0	1	1341	1	1341	0	2682	1341	1375	blastp

Key:

Query:	Query sequence identifier

Database: Database sequence identifier

P: Percent similarity

L: Alignment length

G: Gaps

M: Mismatches

QS: Start in the query sequence

QE: End in the query sequence

DS: Start in the database sequence

DE: End in the database sequence

E: E value

BS: Bit score

QL: Query length

DL: Database length

Me: Method

### Timing Web services

**Figure 2:**
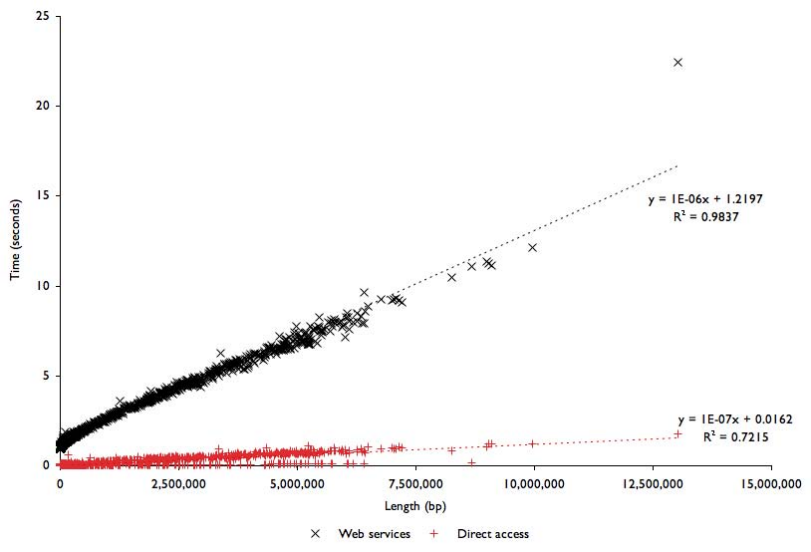
**Timing the SEED Web services**. Time taken to retrieve each complete genome is proportional to the length of the sequence and is limited by network transfer via Web services. The time taken to retrieve each complete genome's sequence either directly from a local SEED installation or via Web services was compared to the length of the DNA sequence. The Web services incurs an approximately ten-fold delay. Linear regression demonstrates that approximately 1,000,000 bp per second are retrieved using the Web services interface.

The major drawback to the Web services approach to computational biology is the significant delay that may be incurred accessing and retrieving data. This is particularly exacerbated in bioinformatics applications where often very many small calls need to be made (e.g. retrieve an identifier or location). Two tests were developed to quantify this delay and provide an estimate of the additional burden of using the Web services interface compared to direct access to a local installation of the API. The Web services were used to access machines at Argonne National Laboratory from San Diego State University, representative of a typical use of Web services to access data. In the first example the DNA sequence was retrieved for each of the complete bacterial genomes in the SEED database, and the time required compared to the length of the sequence. As shown in Fig. 2, access times are linear with respect to sequence length, showing that there is minimal delay in instantiating the backend and accessing the data. The Web services approach takes approximately ten times longer to access the DNA sequence than direct access, but still remain at microseconds per base, placing relatively complex calculations within the realm of realistic computation time. Secondly, a real-world example was used to compare the computational speed using Web services and direct access to the data. The shannon.pl code provided online is the Perl code to compute Shannon's Uncertainty Index [17,18] on intergenic and genic DNA sequences for an entire genome. A minor modification of the script that just redirects the Web services call to a local instantiation (not provided) was used to compute the same analysis with local access to the data. Each calculation was run five times independently on different days and at different times to calculate the uncertainty of intergenic and genic sequences in *V. cholerae*. In each case, more than 15,000 calls were made to the API. Using local access to the API those calls and the computation took 3,409 seconds (±33 seconds; about 1 hour), but using Web services the computation took 21,519 seconds (±144 seconds; about 6 hours). There is an overhead for using the Web services, however the benefit is that there is no need to install or maintain a local copy of the data. Therefore, accessing the data via Web services maybe a more attractive and feasible alternative to maintaining a local installation if rapid computation is not an issue.

## Discussion

Web services provide a mechanism for computational access to the data housed in our databases. The API allows all users to access our systems, retrieve data, and develop tools for mining genomes and metagenomes essentially without restriction. The API provides a flexible interface that has evolved in response to common requests for our end users and will continue to morph in response to demand. The primary advantage of accessing our data via the API is that the data are constantly updated. Although stand-alone SEED installations are available, almost as soon as the installation is complete, it is out of date and needs updating. In contrast, the Web services access data that is mirrored nightly to ensure constant quality and timeliness.

The main drawback with using the Web services approach to access the data rather than via a local installation is the additional overhead associated with transferring the data over the internet. Accessing the data indirectly over a typical internet connection takes about ten times longer than having direct access to the data. However, as the computational processing time increases, those delays are mitigated. On the back-end, the overhead is being mitigated with server-side controls to limit the amount of data transferred. For example, the *search_and_grep *method described here significantly reduces the data returned from database searches. On the front-end prefetching the data, and maintain local caches of limited parts of the data may prove an attractive alternative to continually retrieving large data sets.

In this work to date, we have chosen to implement an RPC/Encoded style of Web service. There are two common Web services approaches: RPC/Encoded and Document/Literal [19]. The general advantage of the former is that it is significantly easier to implement and has a more "natural" style. For example, BLAST search results are returned as tab separated text, just as if they had been computed locally. The disadvantage is that it is much harder for the programmer accessing the data, as they have to individualize each call and the way the data is processed. In contrast, the Document/Literal style uses XML for both the call and response. The XML returned is self-descriptive and self-validating, allowing more automated analysis of the data. Currently we only support the RPC/Encoded style of Web services. In part it was a design decision based on the Perl back end of the SEED API (RPC/Encoded support is conveniently and dynamically supplied by the Perl module POD::WSDL [20]). In addition, this decision allowed us to provide immediate unfettered access to our data while we develop and deploy the more formal Document/Literal style of encoding. We anticipate future releases of the SEED Web services API will move towards Document/Literal even while we continue to support the RPC/Encoded style.

The current SEED API does not limit access in any way. For example, there is no limit on how frequently calls may be made. However, too many repeated calls may be misconstrued as a denial of service (DOS) attack by the host, and therefore users are cautioned to throttle their requests appropriately.

We have provided many code examples both in the Additional File 1 and online at http://ws.theseed.org/. The service is also included in the BioCatalogue http://www.biocatalogue.org/ and future services will also be included there. Users are encouraged to contact the authors to share code and to provide reusable code fragments.

## Conclusions

The SEED family of databases and associated software (Fig. 1), are a comprehensive set of microbial genome annotation and analysis databases. Every microbial genome sequenced to date is stored in these databases, and the annotation servers provide a flexible framework for both complete genomes and metagenomes. Researchers are encouraged to try the programmatic access to the SEED as an alternative means of retrieving data.

## Availability and requirements

• **Project name**: SEED Web services API

• **Project home page**: http://ws.theseed.org/

• **Operating system(s)**: Platform independent

• **Programming language**: Language independent

• **Other requirements**: SOAP

• **License**: SEED Toolkit Public License

• **Any restrictions to use by non-academics**: no limitations

## Abbreviations

FID: (A FIG ID, an internal identifier in the format fig|xxxxxx.i.peg.yyyy); FIG: (Fellowship for Interpretation of Genomes); mg-RAST: (Server for metagenome annotations based in part on RAST technology); RAST: (Server for Rapid Annotations using Subsystem Technology); SEED: (The database and infrastructure for comparative genomics).

## Authors' contributions

TD and RE developed the Web services and wrote most of the example code. SA developed the shannon.pl code and performed the efficiency tests. DC developed and tested the Java code. All authors have contributed to instantiating, testing, and using the services and the underlying databases. All authors contributed to, read, and approved the final version of the manuscript.

## Supplementary Material

Additional file 1 **Example code snippets**. The additional file contains example code in Perl, Python, and Java that demonstrates how to access the SEED using SOAP. Click here for file
