# Supplementary material for: Accessing the SEED Genome Databases via Web Services API: Tools for Programmers
Source: BMC Bioinformatics. 2010 Jun 14;11:319. doi: 10.1186/1471-2105-11-319 (PMC2900279; doi:10.1186/1471-2105-11-319)
Supplement: Additional file 1 — Example code snippets. The additional file contains example code in Perl, Python, and Java that demonstrates how to access the SEED using SOAP. [file 1471-2105-11-319-S1.DOC]

# Example code

These code examples demonstrate the use of SOAP Web services to access the SEED. Examples will compile and run on standard installations of Perl, Python, or Java, and have been tested on Mac OSX and Ubuntu Linux installations.

Code 1:

#!/usr/bin/perl -w

use strict;

use SOAP::Lite;

$service = SOAP::Lite->service('http://ws.theseed.org/FIG/wsdl.cgi');

Code 2:

fig|243277.1.peg.4400

Vibrio cholerae O1 biovar eltor str. N16961 243277

GeneID:2615094 NP_229982.1 VC0328 gi|15640355 gi|41019520 kegg|vch:VC0328 sp|Q9KV30 uni|Q9KV30

DNA-directed RNA polymerase beta subunit (EC 2.7.7.6)

master

Code 3:

Perl

#!/usr/bin/perl -w

use strict;

use SOAP::Lite;

$service = SOAP::Lite->service('http://ws.theseed.org/FIG/wsdl.cgi');

print $service->genomes(‘true’, undef, ‘Bacteria’);

Python:

from SOAPpy import WSDL

service = WSDL.Proxy("http://ws.theseed.org/FIG/wsdl_seed.cgi")

print service.genomes(“true”, ‘’, ‘Bacteria’)

Java:

import java.rmi.RemoteException;

import org.theseed.seed_viewer.SeedWebServices.SeedWebServicesHandler;

import org.theseed.seed_viewer.SeedWebServices.SeedWebServicesHandlerProxy;

public class GenomeProteins {

public static void main(String[] args) {

SeedWebServicesHandler service = new SeedWebServicesHandlerProxy();

String genomes[];

try {

genomes = service.all_genomes("complete", "", "Bacteria");

for (String genome : genomes)

System.out.println(service.genus_species(genome) +

" (" + genome + ")" );

} catch (RemoteException e) {

e.printStackTrace();

System.exit(-1);

}

}

}

Code 4:

$service->adjacent($service->pegs_of('243277.1'));

Code 5:

#!/usr/bin/perl -w

use strict;

use SOAP::Lite;

my $service = SOAP::Lite->service('http://ws.theseed.org/FIG/wsdl.cgi');

my $peg='fig|243277.1.peg.4400';

print "Feature location:\n", $service->feature_location($peg), "\n";

print "Protein sequence:\n", $service->translation_of($peg), "\n";

Code 5 Output:

Feature location: NC_002505_340374_344399

Protein sequence:

>fig|243277.1.peg.4400

MVYSYTEKKRIRKDFGTRPQVLDIPYLLSIQLDSFEKFIEQDPEGQYGLEAAFRSVFPIQSYNGNSELQYVSYRLGEPVFDVKECQIRGVTYSKPLRVKLRLVIFDKDAPAGTVKDIKEQEVYMGEIPLMTENGTFVINGTERVIVSQLHRSPGVFFDSDKG…

(note that the protein sequence has been truncated here, but the full length sequence is returned).

Code 6:

#!/usr/bin/perl -w

use strict;

use SOAP::Lite;

my $service = SOAP::Lite->service('http://ws.theseed.org/FIG/wsdl.cgi');

print $service->sims('fig|243277.1.peg.4400', 5, 1e-5);
